# Synthesis,Antidiabetic and Antitubercular Evaluation of Quinoline–pyrazolopyrimidine hybrids and Quinoline‐4‐Arylamines

**DOI:** 10.1002/open.202400014

**Published:** 2024-03-20

**Authors:** Nosipho Cele, Paul Awolade, Pule Seboletswe, Lungisani Khubone, Kolawole Olofinsan, Md. Shahidul Islam, Audrey Jordaan, Digby F. Warner, Parvesh Singh

**Affiliations:** ^1^ School of Chemistry and Physics University of KwaZulu-Natal, P/Bag X54001, Westville Durban South Africa; ^2^ Department of Biochemistry School of Life Sciences University of Kwazulu-Natal, Westville Durban South Africa; ^3^ Molecular Mycobacteriology Research Unit Department of Pathology and Institute of Infectious Disease and Molecular Medicine University of Cape Town Observatory 7925 South Africa

**Keywords:** quinoline hybrids, pyrazolopyrimidine, α-glucosidase inhibitors, free radical scavengers, antitubercular properties

## Abstract

Two libraries of quinoline‐based hybrids 1‐(7‐chloroquinolin‐4‐yl)‐1*H*‐pyrazolo[3,4–*d*]pyrimidin‐4‐amine and 7‐chloro‐*N*‐phenylquinolin‐4‐amine were synthesized and evaluated for their α‐glucosidase inhibitory and antioxidant properties. Compounds with 4‐methylpiperidine and *para*‐trifluoromethoxy groups, respectively, showed the most promising α‐glucosidase inhibition activity with IC_50_=46.70 and 40.84 μM, compared to the reference inhibitor, acarbose (IC_50_=51.73 μM). Structure‐activity relationship analysis suggested that the cyclic secondary amine pendants and *para*‐phenyl substituents account for the variable enzyme inhibition. Antioxidant profiling further revealed that compounds with an *N*‐methylpiperazine and *N*‐ethylpiperazine ring, respectively, have good DPPH scavenging abilities with IC_50_=0.18, 0.58 and 0.93 mM, as compared to ascorbic acid (IC_50_=0.05 mM), while the best DPPH scavenger is NO_2_‐substituted compound (IC_50_=0.08 mM). Also, compound with *N*‐(2‐hydroxyethyl)piperazine moiety emerged as the best NO radical scavenger with IC_50_=0.28 mM. Molecular docking studies showed that the present compounds are orthosteric inhibitors with their quinoline, pyrimidine, and 4‐amino units as crucial pharmacophores furnishing α‐glucosidase binding at the catalytic site. Taken together, these compounds exhibit dual potentials; *i. e*., potent α‐glucosidase inhibitors and excellent free radical scavengers. Hence, they may serve as structural templates in the search for agents to manage Type 2 diabetes mellitus. Finally, in preliminary assays investigating the anti‐tubercular potential of these compounds, two pyrazolopyrimidine series compounds and a 7‐chloro‐*N*‐phenylquinolin‐4‐amine hybrid showed sub‐10 μM whole‐cell activities against *Mycobacterium tuberculosis*.

## Graphical abstract

This study involves a comparative antidiabetic evaluation, antioxidant and anti‐tubercular evaluation of quinoline‐pyrazopyrimidines and their simple quinoline‐4‐arylamine analogues. The incorporation of pyrazopyrimidine ring to quinoline generally lowers the antidiabetic, antioxidant and antitubercular activity. The molecular docking simulations further discloses these compounds to be the allosteric inhibitors of the α‐glucosidase.

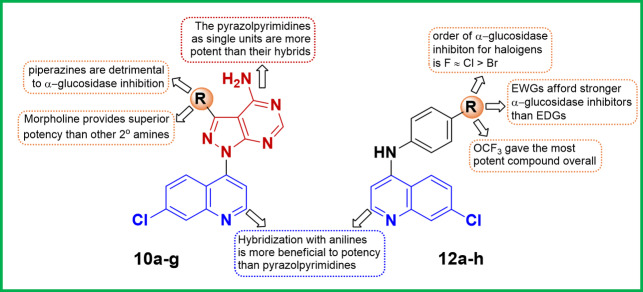

## Introduction

Diabetes mellitus (DM) is a chronic metabolic disorder causing an imbalance in insulin secretion either by its deficiency or resistance; thus, progressing to undesirable high levels of blood glucose that is, hyperglycaemia.[^1^, ^2^] Due to the resulting dysregulation in carbohydrates, fat and protein‐related metabolism, the effects of DM can be fatal if left untreated or poorly managed.^[3]^ Type 2 diabetes mellitus (T2DM), which is caused by insulin resistance, accounts for 90 % of DM cases compared to type 1 diabetes mellitus (T1DM), an autoimmune disorder where the pancreatic cells secrete insufficient levels of insulin.^[4]^ Moreover, complications in patients with T2DM arise from chronic hyperglycemia which may proceed to organs failure and cardiovascular diseases over a period of time.^[5]^ The key therapeutic approach to T2DM management is to regulate the digestion and absorption of carbohydrates and consequently, hyperglycemia. This approach relies on the inhibition of carbohydrate hydrolyzing enzymes such as α‐glucosidase and α‐amylase, hence, they are regarded as crucial molecular targets for T2DM management.

α‐Glucosidase resides in the mucosal brush border of small intestine cells and is significant for the hydrolysis of disaccharides to single glucose sugars and their absorption in the bloodstream.^[6]^ Known α‐glucosidase inhibitors (AGIs) such as acarbose, miglitol and voglibose compete with glucose sugars for binding to the active or the allosteric sites of α‐glucosidase to induce inhibition.^[7]^ The structural similarity of these AGIs to glucose favors a stronger affinity to α‐glucosidase compared to the natural substrate.^[8]^ Consequently, this competitive inhibition reduces α‐glucosidase activity and delays glucose absorption which in turn suppresses postprandial hyperglycemia to relieve T2DM patients.^[9]^ However, the continuous oral administration of these AGIs is associated with adverse effects such as abdominal bloating, diarrhea, and flatulence.^[10]^

Furthermore, the accumulation of reactive oxygen species (ROS) due to glucose auto‐oxidation, decreased nitric oxide bioavailability and non‐enzymatic protein glycation are other critical complications associated with hyperglycemia.^[11]^ Oxidative stress develops when there is a buildup of ROS which overwhelms the cells′ antioxidant defense system during cellular reactions.^[12]^ In mammals, these ROS are produced by enzymatic reactions during ATP biosynthesis and electron and proton transfers for oxygen production.^[13]^ Since oxidative stress is associated with the DM‐induced breakdown of the cellular antioxidant mechanism, antidiabetic drugs with ROS‐scavenging properties are important for preventing complications arising from hyperglycemia.^[14]^ This property is quantifiable using ferric reducing antioxidant power (FRAP), 2,2^’^‐diphenyl‐1‐picrylhydrazyl radical (DPPH) and nitrogen oxide (NO) scavenging assays.^[15]^

The quinoline ring is a known celebrity in medicinal chemistry that is often conjugated with other heterocycles to afford a variety of therapeutic properties such as antitubercular,^[16]^ anticancer,^[17]^ antimalarial,^[18]^ antibacterial^[19]^ and antioxidants.^[20]^ The pharmacophore also helps manage TD2M by initiating the stimulation of insulin secretion through an increase in cAMP which is involved in energy metabolism.^[21]^ The used‐up glucose from energy metabolism then triggers hypoglycemic therapy.^[22]^ Antimalarial drugs such quinine, chloroquine and quinidine are quinoline derivatives known to indirectly trigger hypoglycemic effects and lower glucose levels.^[23]^ The quinoline‐induced hypoglycemic activity effects are also found in gatifloxacin a quinolone displaying a profound blood glucose reduction and directly increasing insulin levels in the pancreas.^[24]^ It is reported that gatifloxacin‐induced hypoglycemia is concomitant to sulfonylurea drugs which are associated with many risk factors for diabetic patients.^[25]^ Numerous quinoline derivatives have also been explored as α‐glucosidase inhibitors.[^1^, ^26^, ^27^]

Recently, because pyrimidines form the core subunit of nucleotides which is vital for RNA and DNA synthesis, medicinal chemistry research is focusing on pyrimidine and its derivatives due to their versatile biological activities such as anti‐inflammatory, antimicrobial, anticancer and antidiabetic.^[28]^ Moreover, Peytam *et al*. have reported the excellent α‐glucosidase inhibitory potentials of pyrimidine hybrids.[^29^, ^30^] Similarly, Pogaku *et al*. reported pyrazole‐triazolopyrimidine hybrids as potent α‐glucosidase inhibitors.^[31]^ Also a naturally occurring amino acid l‐α‐Amino‐β‐(pyrazolyl‐*N*)‐propanoic acid bearing a pyrazole core showed excellent antidiabetic properties^[32]^ even as Kees *et al* reported the antihyperglycemic effects of pyrazole‐containing hybrids on diabetic mice.^[33]^ It is noteworthy that teneligliptin a pyrazole‐containing dipeptidyl peptidase‐4 (DPP‐4) inhibitor is an approved drug for TD2M treatment.^[34]^

Encouraged by these empirical evidences and our previous work on utilizing the molecular hybridization approach to establish new molecular entities with enhanced α‐glucosidase inhibition and antioxidant properties for the treatment of TD2M,^[35]^ we herein report a multistep synthesis for the hybridization of pyrazolopyrimidine core with the quinoline scaffold. Structure‐activity relationship (SAR) was developed by using different secondary amines at the 3‐position of the pyrazole ring. The effect of the pyrazolopyrimidine moiety on bioactivity was also investigated by replacing it with *para*‐substituted anilines. The study findings were then supported by molecular docking calculations to study the interactions of different substituents with α‐glucosidase. Finally, given the interest in both quinolines[^36^, ^37^, ^38^] and pyrazolopyrimidines (39‐40) as potential anti‐tuberculosis agents, the compounds were tested against *Mycobacterium tuberculos*is (Mtb) H_37_Rv in whole‐cell assays.

## Results and Discussion

### Chemistry

**Scheme 1:**
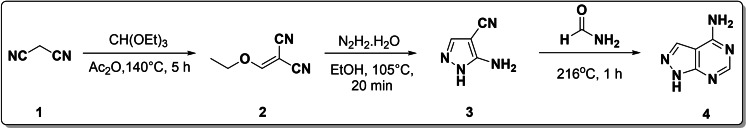
Synthesis of the unsubstituted 4‐aminopyrazolopyrimidine core.

**Scheme 2:**
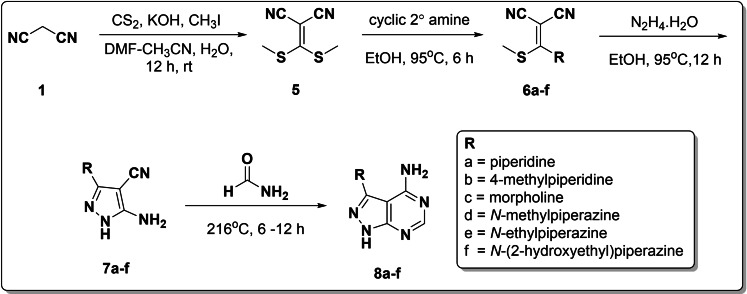
Synthesis of 3‐subsituted pyrazolo[3,4–*d*]pyrimidin‐4‐amine.

**Scheme 3:**
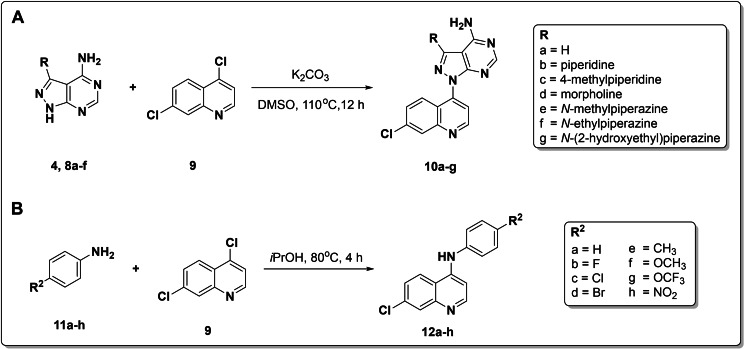
Synthesis of target quinoline hybrids 1‐(7‐chloroquinolin‐4‐yl)‐1*H*‐pyrazolo[3,4–*d*]pyrimidin‐4‐amines and 7‐chloro‐*N*‐phenylquinolin‐4‐amines.

The multistep synthetic route to the target quinoline hybrids is presented in schemes 1, 2–3. Foremost, the unsubstituted 4‐aminopyrazolopyrimidine core was assembled in a three‐step reaction sequence as shown in Scheme 1. The first step is a condensation reaction between triethyl orthoformate and malononitrile **1** to form 2‐(ethoxymethylene)malononitrile **2**. A nucleophilic vinylic substitution reaction or Michael‐type addition of hydrazine hydrate to **2**,[^41^, ^42^] and subsequent intramolecular cyclization yielded 5‐amino‐1*H*‐pyrazole‐4‐carbonitrile **3**. Thereafter, **3** underwent condensation and intramolecular cyclisation in neat formamide to afford 1*H*‐pyrazolo[3,4‐*d*]pyrimidin‐4‐amine **4** as an off‐white solid in quantitative yield (Scheme 1).

Subsequently, to improve the physicochemical properties of 4‐aminopyrazolopyrimidine core and generate a SAR trend for the present library, different cyclic amines were tethered at the 3‐position of pyrazole according to Scheme 2. Malononitrile **1** was subjected to a base‐promoted condensation reaction with carbon disulfide followed by methylation of the potassium thiolate with methyl iodide to form an *S*, *S‐*dimethyl acetal **5**. Treatment of **5** with different cyclic amines followed by condensation with hydrazine hydrate and formamide afforded the desired 3‐substituted 1*H*‐pyrazolo[3,4–*d*]pyrimidin‐4‐amines **8 a**–**f**.

Having constructed the 4‐aminopyrazolopyrimidine cores, the target quinoline‐pyrazolopyrimidine hybrids **10 a**–**g** were synthesized in good yields from the nucleophilic aromatic substitution reaction of 4,7‐dichloroquinoline **9** with either **4** or **8 a**–**f** (Scheme 3a). Additionally, to examine the influence of pyrazolopyrimidine core on biological activity, hybrids **12 a**–**g** were synthesized in a similar manner as **10 a**–**g** using *para*‐substituted anilines (Scheme 3b).

### Structural Elucidation

**Figure 1:**
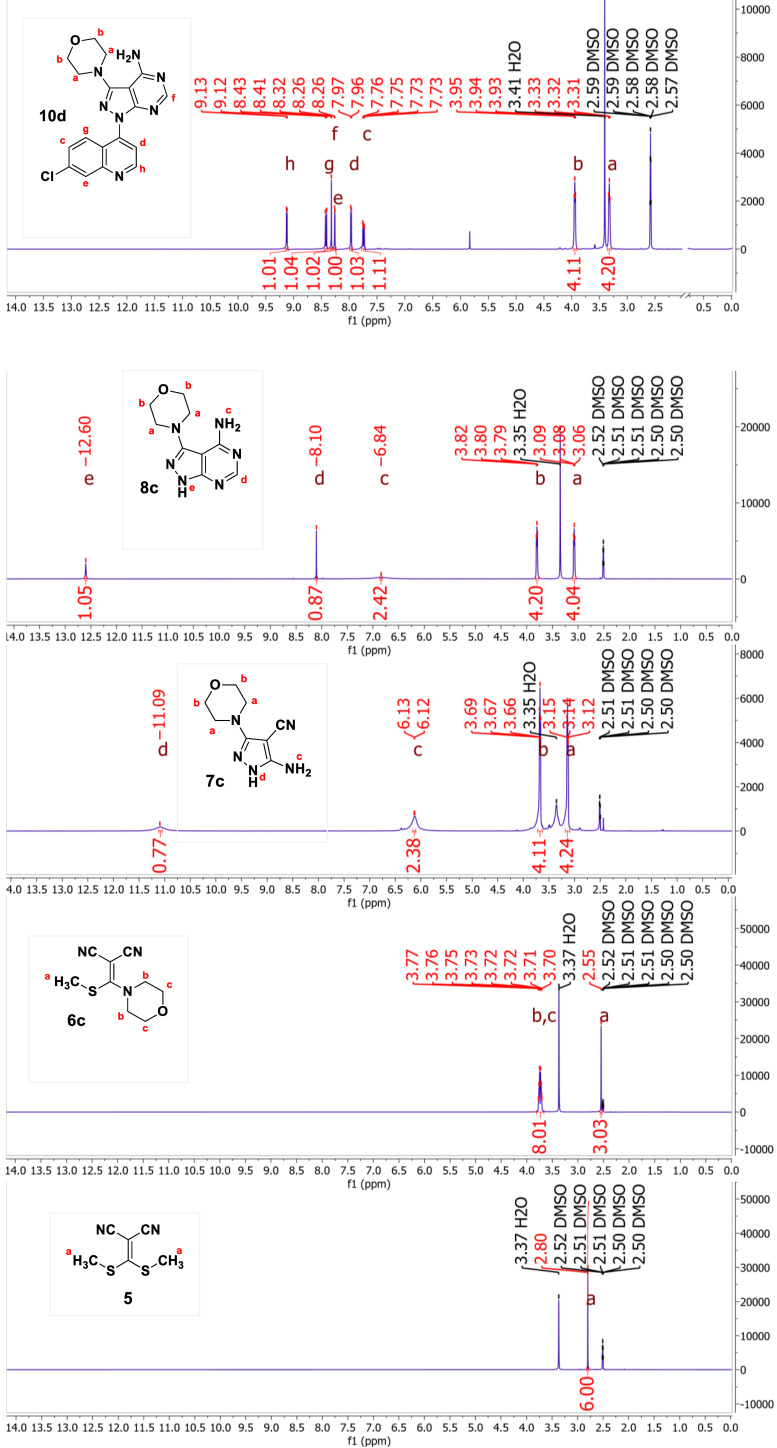
A stack of ^1^H NMR spectra for quinoline‐pyrazolopyrimidine hybrid **10 d** and its precursors.

The quinoline hybrids’ structure was confirmed using 1D (^1^H, and ^13^C) and 2D (HSQC and HMBC) NMR respectively, as well as HRMS. For instance, in the reaction sequence for **10 d**’s synthesis, ^1^H NMR experiments (Figure 1) showed that the tall 6*H* singlet for dithiomethyl groups (δ_H_ 2.80) in intermediate **5** disappeared in the spectrum of **6 c** and emerged as a more shielded 3*H* singlet (δ_H_ 2.55). The presence of an 8*H* multiplet (H_δ_ 3.81‐3.66) also confirmed the tethered morpholine ring. Moreover, the *in situ* cyclization of **6 c** to **7 c** upon treatment with hydrazine hydrate was established by the appearance of two broad singlets corresponding to ‐N*H* (δ_H_ 11.09) and ‐N*H*_2_ (δ_H_ 6.13) protons of the pyrazole ring, respectively. While the cyclization of **7 c** to pyrimidine compound **8 c** was shown by a singlet peak of the pyrimidine ring's proton (δ_H_ 8.10). Finally, the nucleophilic aromatic substitution reaction's success for **10 d**’s formation was evidenced by an m/z 380.1023 in the HRMS spectrum corresponding to[M−H]^+^ and the absence of ‐N*H* proton (δ_H_ 11.09) of pyrazole precursor in the ^1^H NMR spectrum.

**Figure 2:**
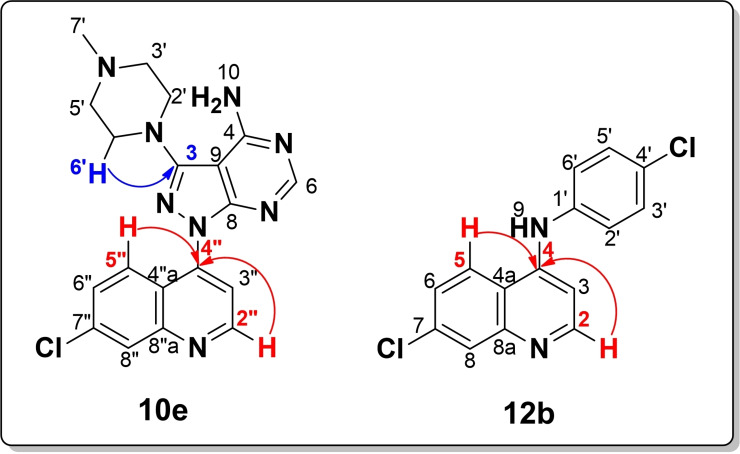
Schematic representation of HMBC correlations in hybrids **10 e** and **12 b**.

The structures of compounds **10 a**–**g** and **12 a**–**h** were confirmed by 2D NMR experiments, *i. e*., heteronuclear multiple bond coherence (HMBC) and heteronuclear single quantum coherence (HSQC) correlations. For example, the HMBC spectrum of **10 e** (Figure 2) featured ^3^ *J* correlations of quinoline H‐2” and H‐5” to C‐4” as well as the ^*3*^ *J* correlation of *N*‐methyl piperazine H‐6’ with C‐3 of pyrazole ring. These correlations confirm the position of each pharmacophore (*i. e*., quinoline, pyrazole and piperazine) in the molecular hybrid. However, no HMBC correlations exist to establish the position of the pyrimidine due to the >^3^ *J* correlation of H‐6’ with quinoline C‐4 quaternary carbon. The same goes for compound **12 b**; phenyl H‐2’ has >^3^ *J* correlation with quinoline C‐4.

### α‐Glucosidase and α‐Amylase Inhibition

The α‐glucosidase and α‐ amylase inhibitory potencies of the quinoline‐pyrazolopyrimidine hybrids (**10 a**–**g**), their precursor (**4**, **8 a**–**f**) and the quinoline‐4‐amine hybrids **12 a**–**h** were determined in an *in vitro* enzyme assay using acarbose as control inhibitor. The IC_50_ values calculated from percentage inhibition are shown in Table 1.

**Table 1:** Inhibitory potency (IC_50_ in μM) of precursor **4**, **8**–**f** and hybrids **10 a**–**g** and **12 a**–**h**.

Entry	R	α‐Glucosidase	α‐Amylase
9		48.43	448.02
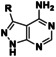			
4	H	57.11	>500
8a	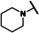	48.15	>500
8b	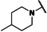	46.70	>500
8c	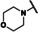	49.53	255.53
8d	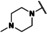	317.17	381.09
8e	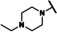	437.05	>500
8f	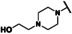	247.77	>500
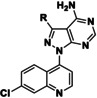			
10a	H	64.69	>500
10b	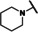	87.22	>500
10c	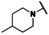	>500	363.23
10d	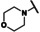	58.48	481.86
10e	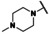	74.40	473.41
10f	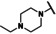	209.94	>500
10 g	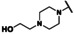	112.86	494.09
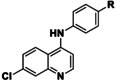			
12a	H	44.22	333.15
12b	F	43.32	>500
12c	Cl	41.45	>500
12d	Br	76.70	407.03
12e	CH_3_	81.13	>500
12f	OCH_3_	60.23	>500
12 g	OCF_3_	40.84	>500
12 h	NO_2_	45.99	>500
Acarbose		51.73	234.05

The results show that α‐glucosidase inhibitory potency is controlled by the nature of cyclic amine pendant on pyrazole. Compounds **8 a**–**c** (IC_50_=46.70‐49.53 μM) are more potent α‐glucosidase inhibitors than the unsubstituted compound **4** (IC_50_=57.11 μM). More importantly, compounds **8 a**–**c** are superior inhibitors compared to acarbose (IC_50_=51.73 μM). The piperazine‐based compounds **8 d**–**f** however showed several fold inferior activities compared to **4** and **8 a**–**c**, thus suggesting that increased basicity is detrimental to potency. Furthermore, the potency of parent quinoline **9** (IC_50_=48.43 μM) and the pyrazolopyrimidines motivated us to molecularly hybridize both pharmacophores to give **10 a**‐**g**. Surprisingly, the approach gave a mixed outcome; a generally reduced α‐glucosidase inhibition for compounds **10 a**–**d** relative to their precursors **4** and **8 a**–**c**, an abolished potency in **10 c** (IC_50_=>500 μM) compared to **8 b**, and an improved potency in piperazine compounds **10 e**–**g** as compared to their precursors (**8 d**–**f**).

To continue our SAR exploration, the pyrazolopyrimidine moieties were replaced with *para*‐substituted anilines in compounds **12 a**–**h** while conserving the quinoline backbone. The focus on *para*‐position stems from our previous study showing *para*‐substituted quinoline‐oxadiazole hybrids as superior α‐glucosidase inhibitors than the *ortho*‐ and *meta*‐substituted analogues.^[35]^ Interestingly, these aniline analogues showed improved activities compared to the pyrazolopyrimidines (**4**, **8 a**–**g**) and pyrazolopyrimidine‐quinoline molecular hybrids (**10 a**–**g)**. These compounds (except for **12 d**–**f**) were also more potent (IC_50_=40.84‐45.99 μM) than acarbose; the OCF_3_‐substituted compound **12 g** (IC_50_=40.84 μM) being the most potent α‐glucosidase inhibitor overall. The superior potency of **12 g** can be attributed to the strong metabolic stability and improved solubility induced by the fluorine atom as well as the excellent conformational stability of the OCF_3_ unit, which enhances binding affinity to drug targets.^[43]^

**Figure 3:**
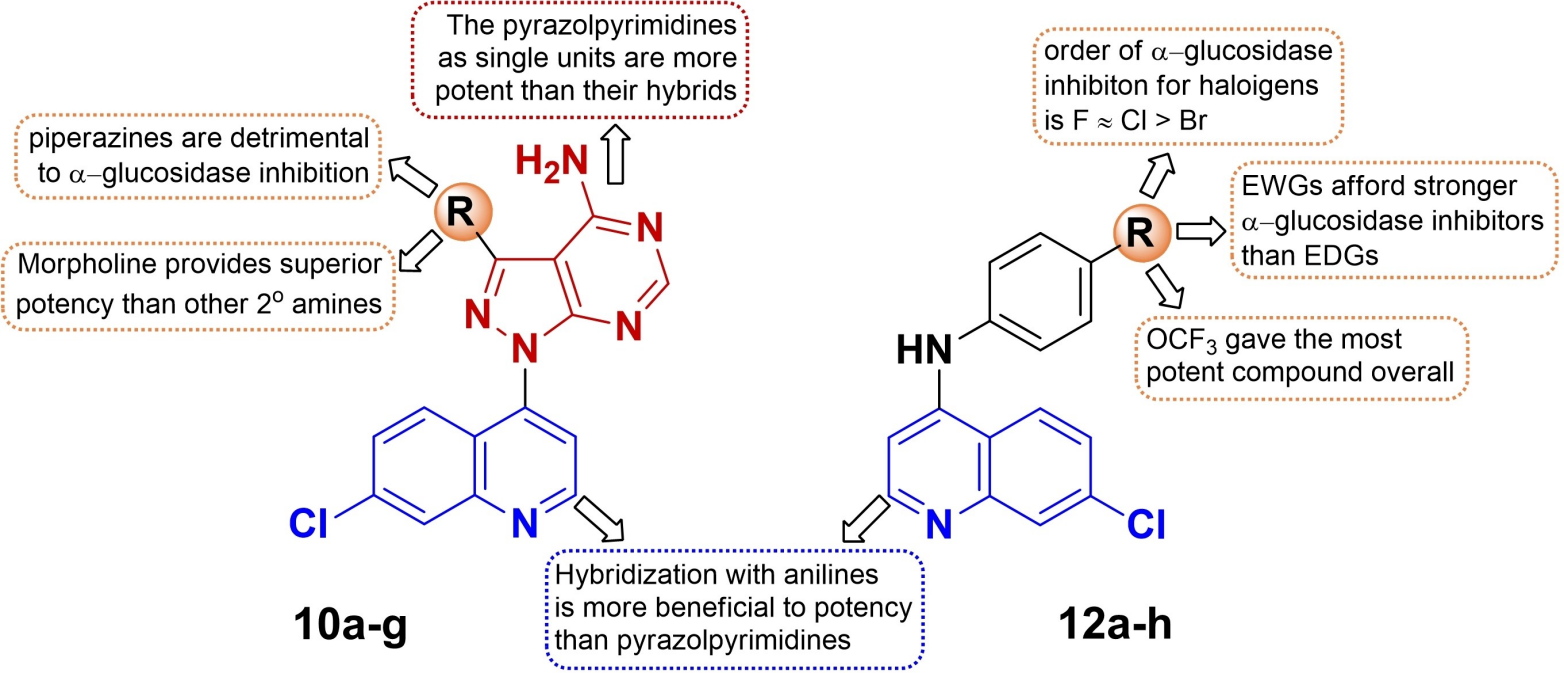
SAR summary of α‐glucosidase inhibitory profile of **10 a**–**g** and **12 a**–**h**.

The SAR analysis of the **12 a**–**h** series also highlighted the effect of substituent type and electronic nature on α‐glucosidase inhibition. Compounds **12 g** (IC_50_=40.84 μM**)** and **12 h** (IC_50_=45.99 μM) bearing electron‐withdrawing substituents were found to be more potent inhibitors than compounds **12 e (**IC_50_=81.13 μM**)** and **12 f** (IC_50_=60.23 μM) bearing electron‐donating substituents. A similar SAR exists in halogenated compounds **12 b**–**d** for which the order of α‐glucosidase inhibition (F≈Cl>Br) varies linearly with the halogen's electronegativity and increase in atomic radius such that the bigger size of Br prevents the compound to properly fit in the active site of the target. Interestingly, this SAR trend is on a par with our previous study.^[35]^ Hence, it is affirmable that electron‐withdrawing groups, particularly fluorinated units are crucial for strong α‐glucosidase inhibition. In contrast, the present compound series exhibited poor α‐amylase inhibition, a property inherited from the parent quinoline compound **9**; hence, a distinctive SAR cannot be established. A summary of existing SAR for α‐glucosidase inhibition is shown in Figure 3.

### 1 Antioxidant Activity

To assess the present compounds’ ability to mitigate hyperglycaemia‐induced ROS accumulation and corresponding complications in T2DM patients, the compounds were evaluated for their antioxidant activity *in vitro* using DPPH and NO radical scavenging assays. The results are presented in Table 2.

**Table 2:** Antioxidant profile (IC_50_ in mM) of precursor **4**, **8 a**–**f** and hybrids **10 a**–**g** and **12 a**–**h**.

Entry	R	DPPH	NO
9		>4	0.82
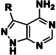			
4	H	>4	0.78
8a	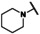	3.40	0.80
8b	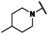	>4	1.01
8c	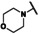	>4	0.88
8d	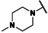	0.58	0.80
8e	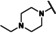	0.93	0.98
8f	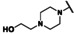	3.54	0.60
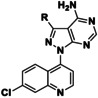			
10a	H	>4	2.59
10b	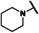	>4	>4
10c	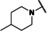	2.10	2.78
10d	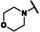	>4	2.46
10e	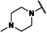	>4	>4
10f	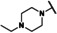	>4	0.80
10 g	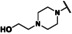	2.96	0.28
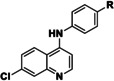			
12a	H	>4	0.50
12b	F	>4	1.92
12c	Cl	>4	>4
12d	Br	>4	>4
12e	CH_3_	>4	>4
12f	OCH_3_	>4	0.88
12 g	OCF_3_	>4	>4
12 h	NO_2_	0.08	0.65
Ascorbic acid		0.05	>4

***DPPH scavenging activity***. A comparison of the DPPH scavenging ability of parent quinoline **9** (IC_50_=>4 mM) with pyrazolopyrimidines (**8 a**–**f)** and their molecular hybrids with quinoline (**10 a**–**g)** including the arylamine‐substituted quinolines (**12 a**–**h)** revealed that molecular hybridization only improved the antioxidant profile in hybrids **10 c** (IC_50_=2.10 mM) and **10 g** (IC_50_=2.96 mM) while other quinoline hybrids replicated the inferior DPPH scavenging activity of parent compound **9**. Notably, compound **12 h** emerged as the best DPPH scavenger with an IC_50_ value of 0.08 mM akin to the reference antioxidant, ascorbic acid (IC_50_ of 0.05 mM). Other promising DPPH scavengers are pyrazolopyrimidine **8 a**, **8 d**, **8 e**, and **8 f** with IC_50_=3.40, 0.58, 0.93 and 3.54 mM, respectively. The results indicate that the inactive compounds do not possess enough electron density to stabilize electron transfer for DPPH radical scavenging.^[44]^

***NO scavenging activity***. In contrast to the DPPH assay, the NO scavenging ability of the compounds is generally promising, akin to parent compound **9** (IC_50_=0.82 mM). Amidst the pyrazolopyrimidines, **4** and **8 f** were the best NO scavengers with IC_50_=0.78 and 0.60 mM, respectively. Interestingly, the significance of molecular hybridization was most highlighted in quinoline‐pyrazolopyrimidine hybrid **10 g** bearing a *N*‐(2‐hydroxyethyl)piperazine moiety. With an IC_50_ of 0.28 mM, the compound has ~2‐ and 3‐fold enhanced NO scavenging activity compared to parent compounds **8 f** (IC_50_=0.60 mM) and **9** (IC_50_=0.82 mM), respectively. The improvement was also replicated in compound **10 f** (IC_50_=0.80 mM) and quinoline‐4‐arylamines **12 a** and **12 h** with IC_50_=0.50 and 0.65 mM, respectively. Overall, compounds **8 a**, **10 g**, and **12 h** possess the best antioxidant profile (DPPH and NO radical scavenging abilities) in the present library.

## Molecular Modelling

Docking calculations were performed to understand the molecular level interactions of the most potent α‐glucosidase inhibitors *viz*., compounds **8 b**, **10 d**, and **12 g**, and consequently, unearth the pharmacophore features driving enzyme inhibition. The *in silico* investigation was also explored to predict the compounds’ mode of inhibition using induced fit docking at the active (or orthosteric) and allosteric sites, respectively. The allosteric site partially overlaps the active site as previously studied by Taha *et al* ^[45]^ and in our previous work.^[35]^ The results shown in Table 3 suggest that the compounds have stronger and more stable binding affinities for the α‐glucosidase active site as compared to the allosteric site; hence, they can be regarded as active site inhibitors and not allosteric inhibitors.

**Table 3:** Results of IFD and MMGBSA binding free energy (ΔG_bind_) calculations.

Compounds	Docking score	Glide emodel	Glide energy	ΔG_bind_ (kcal.mol^−1^)
Orthosteric site				
8b	−6.421	−72.496	−49.402	−72.13
10d	−6.757	−65.807	−48.445	−62.79
12 g	−8.133	−67.164	−46.226	−71.33
acarbose	−11.994	−57.848	−56.889	−90.55
Allosteric site				
8b	−6.490	−25.866	−19.500	−35.54
10d	−6.904	−36.406	−24.563	−17.13
12 g	−4.373	−25.589	−20.419	−36.94

**Figure 4:**
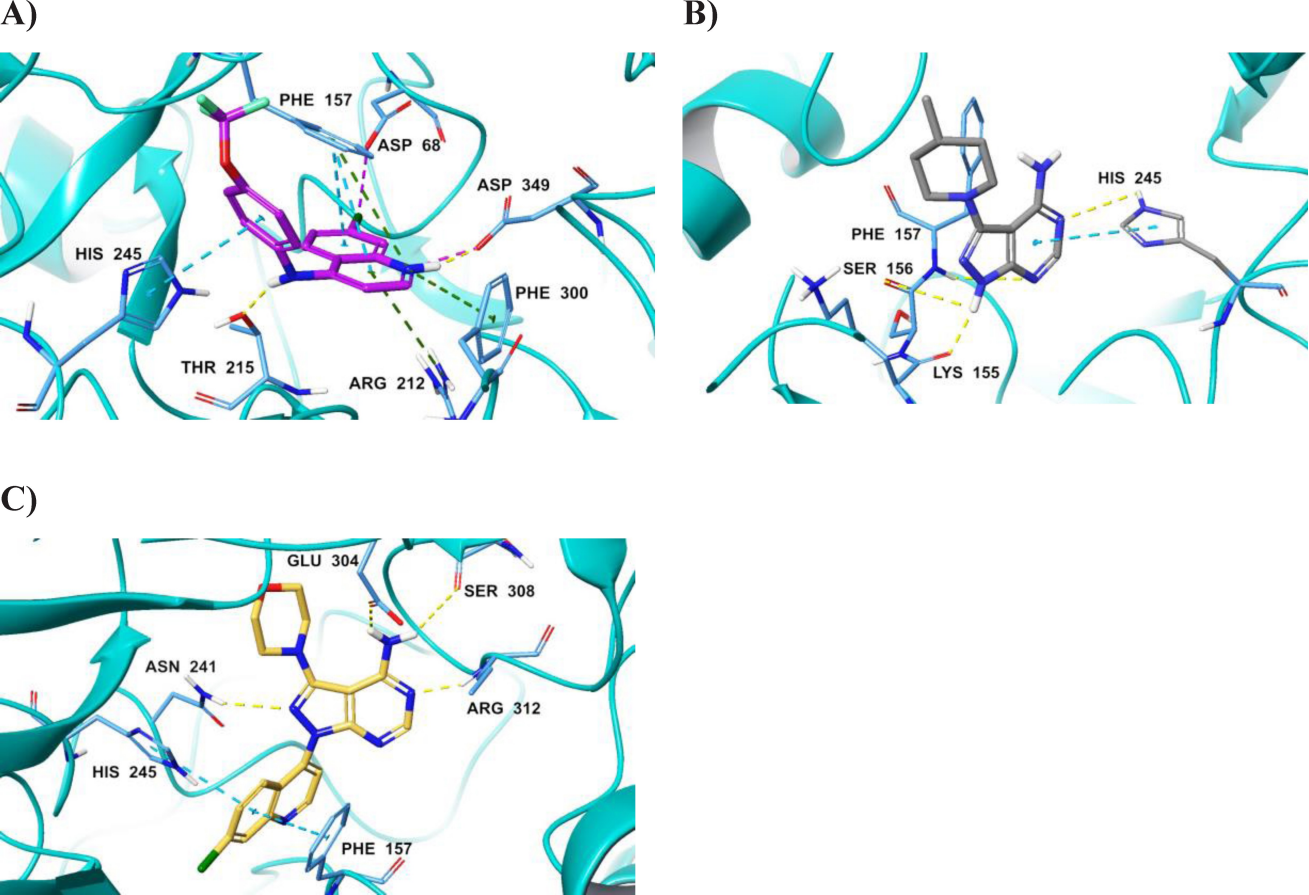
3‐D representation of the ligand‐protein interactions in compound **12 g** (**A**), **8 b** (**B**) and **10 d** (**C**).

The binding profile in compound **12 g**‐α‐glucosidase complex (Figure 4A) highlights the ligand's perfect fit at the binding site as depicted by the highest docking score (−8.133) and binding free energy (ΔG_bind_=‐71.91 kcal.mol^−1^). Thus, validating its enhanced α‐glucosidase inhibition compared to **8 b** and **10 d**.

The ligand‐protein interactions in **12 g**‐α‐glucosidase complex includes hydrogen bond (H‐bond) interaction of 4‐amino unit with Thr215, similar H‐bond and electrostatic interactions of the protonated quinoline nitrogen with Asp349 and π‐cation interactions of the quinoline core with Phe157, Arg212 and Phe300. The quinoline's 7‐chloro unit also formed halogen bond interaction with Asp68 while the quinoline and phenyl rings interacted *via* π‐π stacking interactions with Phe137 and His245, respectively.

The biochemical data showed that pyrazolopyrimidine compound **8 b** exhibited the second highest inhibitory activity; hence, **8 b**‐α‐glucosidase complex (Figure 4B) showed a docking score of ‐6.421 and ΔG_bind_ of ‐72.13 kcal.mol^−1^. The higher ΔG_bind_ of this compound compared to compounds **12 g** and **10 d** indicates a stronger affinity to the active site. The pyrazole NH in compound **8 b** served as an H‐bond donor to Lys155 and Ser156 while the pyrimidine nitrogen atoms were H‐bond acceptors to Phe157 and His245. The pyrimidine ring also formed π‐π stacking interaction with His245. The absence of H‐bond and electrostatic interactions with conserved catalytic residue Asp349 in **8 b**‐α‐glucosidase complex as seen in compound **12 g** presumably accounts for the slightly reduced potency of the former.

Although compound **10 d** showed a lower inhibitory potency relative to compounds **8 b** and **12 g**, the docking score (−6.757) was comparable to **8 b** albeit the ΔG_bind_ of −62.79 kcal.mol^−1^ was lower than **8 b** and **12 g** complexes. Analysis of compound **10 d**‐α‐glucosidase complex (Figure 4C) revealed H‐bond interactions of; pyrazolo nitrogen with Asn241, pyrimidine nitrogen with Arg312 and 4‐amino unit with Glu304 and Ser308, respectively. Notably, the quinoline core did not participate in strong H‐bond and electrostatic interactions as seen in **12 g**, instead, it formed π‐π stacking interactions with Phe157 and His245 even as the halogen bond interaction of 7‐chloro unit is absent. Conceivably, these dissimilarities in the interaction profile account for the reduced α‐glucosidase inhibition between **10 d** and **12 g**.

## Anti‐Mtb Activity in Vitro

The antimycobacterial potential of compounds (**4**,**9**,**8 a**–**12 h**) was evaluated during aerobic culture of *Mtb* H_37_Rv for seven to fourteen days in two different growth media used routinely to determine the possible impact of protein binding on activity (Table 4). Compared to the Rifampicin control, the MIC_90_ values of most compounds indicated weak to no anti‐*Mtb* activity in these assays. Notable exceptions were the pyrazolopyrimidine series compounds **8 b** and **8 c** with piperidine and 4‐methylpiperidine secondary amines as R groups, which showed excellent activity with MIC_90_=7.748 μM and MIC_90_=7.149 μM, respectively. Moderate activity was also witnessed in compounds **8 d** and **8 e** with morpholine and *N*‐methylpiperazine in 14 days. When 4,7‐dichloquinoline was hybridized with pyrazolopyrimidine furnished with *N*‐methylpiperazine, good activity was evident as displayed in compound **10 e** with MIC_90_=31.25 μM in 7 days. This activity was also moderately demonstrated in compounds **10 f** and **10 g** containing *N*‐ethylpiperazine and *N‐*(2‐hydroxyethyl piperazine) groups. These findings suggest that the common piperazine group in the latter three compounds promotes the activity. Moreover, when the pyrazolopyrimidine moiety is substituted with simple *para*‐anilines, activity was elevated, as shown in **12 b** and **12 g** MIC_90_=31.25 μM and MIC_90_=9.904 μM, compounds having a common flouro substituent in the *para* position. In conclusion, direct hybridization of the quinoline to the pyrazolopyrimidine conferred moderate anti‐*Mtb* activity but when the pyrazolopyrimidine was substituted with a piperazine group the activity was elevated.

**Table 4:** Compounds **4‐12 h** 90 % *Mtb* inhibition in 7H9 ADC GLU Tw and 7H9 CAS GLU Tx media.

	7H9 CAS GLU Tx		7H9 ADC GLU Tw	
Compound ID	GFP Assay – 10pt DR: MIC90 (uM) [Media: 7H9 CAS GLU Tx, Day:7]	GFP Assay – 10pt DR: MIC90 (uM) [Media: 7H9 CAS GLU Tx, Day:14]	GFP Assay – 10pt DR: MIC90 (uM) [Media: 7H9 ADC GLU Tw, Day:7]	GFP Assay – 10pt DR: MIC90 (uM) [Media: 7H9 ADC GLU Tw, Day:14]
9	>125	>125	>125	>125
4	>125	31.25	>125	>125
8a	>125	125	>125	>125
8b	>125	7.748	>125	>125
8c	>125	7.149	>125	>125
8d	>125	62.5	>125	>125
Rifampicin	0.010	0.014	0.001	0.002
8e	>125	62.5	>125	>125
8f	>125	>125	>125	>125
10a	>125	>125	>125	>125
10b	>125	>125	>125	>125
10c	62.5	>125	125	>125
10d	>125	>125	>125	>125
Rifampicin	0.019	0.010	0.001	0.002
10e	31.25	>125	125	>125
10f	62.5	>125	125	>125
10 g	62.5	62.5	>125	>125
12a	125	62.5	>125	>125
12b	31.25	>125	125	>125
12c	>125	>125	>125	>125
12d	>125	>125	>125	>125
Rifampicin	0.016	0.010	0.001	0.002
12e	31.25	>125	125	>125
12f	62.5	>125	>125	>125
12 g	9.904	62.5	125	>125
12 h	>125	62.5	>125	>125
Rifampicin	0.018	0.016	0.001	0.002

## Conclusions

In this study, compounds containing quinoline and pyrazolopyrimidine heterocycles in their structural framework were tested for their α‐glucosidase and α‐amylase inhibitory potencies and antioxidant properties. The pyrazolopyrimidines **8 a**–**8 c** exhibited superior α‐glucosidase inhibition compared to the control, acarbose. Also, the adopted molecular hybridization strategy enhanced the α‐glucosidase inhibitory potency of parent quinoline compound **9** as seen in OCF_3_‐substituted analogue **12 g**, the most potent α‐glucosidase inhibitor overall. Although the approach was detrimental in the pyrazolopyrimidines, compound **10 c** displayed an inhibitory potency comparable to acarbose. The antioxidant profiling also showcased **8 a**, **10 g** and **12 h** as excellent DPPH and NO radical scavengers. In contrast, the poor α‐amylase inhibition of the present library requires further study to improve their dual‐target efficacy. Molecular docking studies also revealed the significance of quinoline, pyrimidine and 4‐amino units to α‐glucosidase inhibition and highlighted the potent compounds as active (or orthosteric) site inhibitors. Taken together, the compounds identified herein hold the potential for developing new α‐glucosidase inhibitors for T2DM management. Finally, in preliminary anti‐*Mtb* studies, the pyrazolopyrimidines (**8 b** and **8 c)** and the 7‐chloro‐*N*‐phenylquinolin‐4‐amine quinolone hybrid (**12 g**) exhibited potent activity under standard growth conditions *in vitro*, suggesting the potential to explore these compounds further.

## Experimental

### Synthetic Chemistry

The reactions’ progress was monitored *via* thin layer chromatography (TLC) performed on Merck Kiesegel 60 F_254_ plates and visualized under 254 nM UV light. Melting points were determined using a closed‐end capillary tube and are uncorrected. Product purification was achieved with silica gel (0.063–0.200 mm) column chromatography at different polarities of ethyl acetate‐hexane or ethyl acetate‐methanol eluents. ^1^H, ^13^C and 2D‐NMR spectra were recorded on Bruker Avance^III^ 400 and 600 MHz spectrometers. The chemical shifts (δ) in ppm were measured with deuterated dimethyl sulfoxide‐*d*_6_ (δ_H_ 2.50 and δ_C_ 39.50 ppm) downfield with respect to tetramethylsilane (TMS) as internal standard at δ_H_=0. Coupling constants *J* are reported in Hertz (Hz) while the splitting patterns are abbreviated as singlet (s), doublet (d), broad doublet (db), triplet (t), quartet (q), multiplet (m), doublet of doublet (dd), doublet of triplet (dt), triplet of doublet (td), doublet of quartet (dq) and pentet (p). High‐resolution mass spectra were recorded on Water Micromass LCT Premier TOF‐MS spectrometer.

### Synthesis of 1*H*‐Pyrazolo[3,4–*d*]pyrimidin‐4‐Amine, 4

Acetic anhydride (20 mL) was added to a mixture of malononitrile **1** (5.80 g, 87.80 mmol) and triethyl orthoformate (22 mL, 13 mmol) and then refluxed at 140 °C for 5 h. The reaction was cooled to room temperature (rt), then acetic acid was removed under reduced pressure. The residue was cooled to r.t and poured into an ice slurry while stirring. The precipitated yellow crystals of 2‐(ethoxymethylene)malononitrile **2** weighing 9.29 g were collected *in vacuo* filtration and washed with water. Subsequently, compound **2** (5.00 g 40.94 mmol) was dissolved in absolute ethanol and hydrazine hydrate was carefully added at 0 °C while stirring, followed by reflux at 105 °C for 20 minutes. After the reaction was complete as monitored by TLC, the mixture was cooled and poured into an ice slurry and the precipitate formed was filtered and dried under vacuum to afford compound **3** as a yellow‐orange solid weighing 2.47 g. Compound **3** (2.46 g, 18.20 mmol) was added to formamide (7 mL) and vigorously stirred under reflux at 216 °C for 45 minutes. The resulting precipitate was cooled to r.t, poured in an ice slurry and filtered *in vacuo* to give 2.42 g of 1*H*‐pyrazolo[3,4–d]pyrimidin‐4‐amine **4** as a cream precipitate with 54.57 % yield over three steps.

#### 5‐Amino‐1H‐Pyrazole‐4‐Carbonitrile (3)

Yellow‐orange solid; Chemical formula: C_4_H_4_N_4_; Yield; 78.23 %, Mol. wt: 108.10 gmol^−1^.

^**1**^ **H NMR** (400 MHz, DMSO‐d_6_) δ 11.95 (s, 1H, N*H*), 7.71 (s, 1H, H‐3), 6.01 (s, 2H, N*H*_2_).

#### 1H‐Pyrazolo[3,4–d]pyrimidin‐4‐Amine (4)

Cream solid; Chemical formula: C_5_H_5_N_5_; Yield; 71.94 %, Mol wt: 135.13 gmol^−1^.

^**1**^ **H NMR** (400 MHz, DMSO‐d_6_) δ 13.34 (s, 1H, N*H*), 8.14 (s, 1H, H‐3), 8.08 (s, 1H, H‐6), 7.60 (bs, 2H, N*H*_2_).

^**13**^ **C NMR** (101 MHz, DMSO‐d_6_) δ 158.47 (C‐3a), 156.28 (C‐3), 155.27 (C‐7a), 133.04 (C‐6), 100.06 (C‐4).

**HRMS**: (ESI^+^‐MS, *m*/*z*) calcd for C_5_H_5_N_5_ (M−H)^+^: 134.0467; found: 134.0468.

### General Synthesis of Pyrazolopyrimidines 8 a–g

A solution of malononitrile (13.5 g, 204.4 mmol), potassium hydroxide (22.05 g, 392.9 mmol) and DMF:H_2_O (1 : 1.4) in acetonitrile was stirred in an ice bath (0–5 °C) for 20 minutes, then carbon disulfide (12.6 mL, 165.5 mmol) was added in drops and the solution was stirred for 3 hours at r.t. Methyl iodide 25.5 mL was added and stirring continued overnight. The mixture was then poured into crushed ice and vigorously stirred to form a precipitate which was filtered and washed with cold water to give compound **5** as a yellow solid (27.17 g, 96.79 % yield). Thereafter, appropriate cyclic secondary amines (11.75 mmol) and compound **5** (11.75 mmol) were dissolved in absolute ethanol and refluxed at 105 °C for 6 hours. After *in vacuo* evaporation of ethanol, the residue was cooled in the freezer overnight and the resulting crystals were collected by vacuum filtration while washing with cold ethanol to give compound **6 a**–**f** in excellent yields.

Compounds **6 a**–**f** (7.65 mmol) were then dissolved in absolute ethanol and treated with hydrazine hydrate (7.64 mmol) at 0 °C, followed by reflux at 105 °C for 12 hours. After cooling to r.t and crystallizing in the fridge overnight, the resulting crystals were collected by vacuum filtration while washing with cold ethanol to afford 5‐amino‐1*H*‐pyrazole‐4‐carbonitriles compound **7 a**–**f** in excellent yields. Finally, compound **7 a**–**f** (4.72 mmol) was vigorously stirred with formamide (4 mL) under reflux at 216 °C for 6–12 hours. After cooling to r.t, the solution was poured into an ice slurry to precipitate the product or concentrated on silica gel and purified by column chromatography using ethyl acetate‐methanol as eluent.

#### 2‐(bis(methylthio)methylene)malononitrile (5)

Deep orange solid; Chemical formula: C_6_H_6_N_2_S_2_; Yield; 96.79 %, Mol wt: 170.26 gmol^−1^.

^**1**^ **H NMR** (400 MHz, DMSO‐d_6_) δ 2.80 (s, 6H, H‐1).

^**13**^ **C NMR** (101 MHz, DMSO‐d_6_) δ 186.45 (C‐1a), 113.77 (C‐2a), 19.36 (C‐1) (Note: some quaternary carbon peaks were not observed).

#### 2‐((methylthio)(morpholino)methylene)malononitrile (6 c)

Cream white solid; Chemical formula: C_9_H_11_N_3_OS; Yield; 80.01 %, Mol wt: 209.27 gmol^−1^.

^**1**^ **H NMR** (400 MHz, DMSO‐d_6_) δ 3.76 (t, *J*=5.0 Hz, 4H, H‐3,4), 3.72 (t, *J*=3.4 Hz, 4H, H‐2,5), 2.55 (s, 3H, H‐1).

^**13**^ **C NMR** (101 MHz, DMSO‐d_6_) δ 176.10 (C‐1), 66.35 (C‐2,5), 53.55 (C‐3,4), 18.24 (C‐1).

#### 5‐Amino‐3‐(piperidin‐1‐yl)‐1H‐Pyrazole‐4‐Carbonitrile (7 a)

Yellow solid; Chemical formula: C_9_H_13_N_5_; Yield; 82.11 % Mol wt: 191.23 gmol^−1^.

^**1**^ **H NMR** (400 MHz, DMSO‐d_6_) δ 11.02 (s, 1H), 6.16 (s, 2H), 3.39 (s, 4H), 1.66–1.56 (m, 6H).

^**13**^ **C NMR (**101 MHz, DMSO‐d_6_) δ 148.52, 119.89, 117.15, 48.98, 25.21, 24.27.

#### 5‐Amino‐3‐(4‐Methylpiperidin‐1‐yl)‐1H‐Pyrazole‐4‐Carbonitrile (7 b)

Cream solid; Chemical formula: C_10_H_15_N_5_; Yield; 76.22 %, Mol wt: 205.26 gmol^−1^.

^**1**^ **H NMR** (400 MHz, DMSO‐d_6_) δ 10.98 (s, 1H, H‐1), 6.05 (s, 2H, H‐5a), 3.69 (dt, *J*=12.8, 3.4 Hz, 2H, H‐2’), 2.66 (s, 2H, H‐6’), 1.61 (dd, *J*=12.9, 3.4 Hz, 2H, H‐3’), 1.52–1.43 (m, 1H, H‐3’a), 1.17 (qd, *J*=12.2, 4.0 Hz, 2H, H‐5’), 0.91 (d, *J*=6.5 Hz, 3H, H‐7’).

^**13**^ **C NMR** (101 MHz, DMSO‐d_6_) δ 145.42, 117.13, 62.75, 48.34, 43.74, 33.47, 30.75, 30.60, 22.28.

#### 5‐Amino‐3‐Morpholino‐1H‐Pyrazole‐4‐Carbonitrile (7 c)

Yellow solid; Chemical formula: C_8_H_11_N_5_O; Yield; 94.89 %, Mol wt: 193.21gmol^−1^.

^1^H NMR (400 MHz, DMSO‐d_6_) δ 11.09 (s, 1H, H‐1), 6.12 (s, 2H, H‐5a), 3.67 (t, *J*=4.8 Hz, 4H, H‐3’,5’), 3.14 (t, *J*=4.8 Hz, 4H, H‐2’,6’).

^**13**^ **C NMR** (101 MHz, DMSO‐d_6_) δ 154.74, 116.85, 66.04, 48.44 (Note: some quaternary carbon peaks were not observed).

#### 5‐Amino‐3‐(4‐Methylpiperazin‐1‐yl)‐1H‐Pyrazole‐4‐Carbonitrile (7 d)

Light yellow solid; Chemical formula: C_9_H_14_N_6_; Yield; 67.91 %, Mol wt: 206.25 gmol^−1^

^**1**^ **H NMR** (400 MHz, DMSO‐d_6_) δ 11.06 (s, 1H, H‐1), 6.04 (s, 2H, H‐5a), 3.16 (t, *J*=5.0 Hz, 4H, H‐2’,6’), 2.38 (t, *J*=4.9 Hz, 4H, H‐3’,5’), 2.18 (s, 3H, H‐7’).

^**13**^ **C NMR** (101 MHz, DMSO‐d_6_) δ 154.92, 116.90, 62.79, 54.38, 47.86, 46.22.

#### 5‐Amino‐3‐(4‐Ethylpiperazin‐1‐yl)‐1H‐Pyrazole‐4‐Carbonitrile (7 e)

Light yellow solid; Chemical formula: C_10_H_16_N_6_; Yield; 71.36 %, Mol wt: 220.27 gmol^−1^.

^**1**^ **H NMR** (400 MHz, DMSO‐d_6_) δ 5.94 (s, 2H, H‐5a), 3.18 (t, *J*=5.0 Hz, 4H, H‐2’&6’), 2.43 (dd, *J*=6.6, 3.5 Hz, 4H, H‐3’,5’), 2.34 (q, *J*=7.2 Hz, 2H, H‐7’), 1.00 (t, *J*=7.2 Hz, 3H, H‐8’).

^**13**^ **C NMR** (101 MHz, DMSO‐d_6_) δ 157.34, 154.90, 116.90, 62.80, 52.12, 47.99, 12.26 (some quaternary carbon peaks were not observed).

#### 5‐Amino‐3‐(4‐(2‐Hydroxyethyl)piperazin‐1‐yl)‐1H‐Pyrazole‐4‐Carbonitrile (7 f)

Light yellow solid; Chemical formula: C_10_H_16_N_6_O; Yield; 69.95 % Mol wt: 236.27 gmol^−1^.

^**1**^ **H NMR** (400 MHz, DMSO‐d_6_) δ 11.01 (s, 1H, H‐1), 6.03 (s, 2H, H‐5a), 3.51 (t, *J*=6.2 Hz, 2H, H‐7’), 3.17–3.14 (m, 4H, H‐2’,6’), 2.49 (q, *J*=4.8 Hz, 4H, H‐3’,5’), 2.41 (t, *J*=6.2 Hz, 2H, H‐8’).

^**13**^ **C NMR** (101 MHz, DMSO‐d_6_) δ 154.84, 117.00, 60.71, 58.88, 52.94, 47.99 (some quaternary carbon peaks were not observed).

#### 3‐(piperidin‐1‐yl)‐1H‐Pyrazolo[3,4–d]pyrimidin‐4‐Amine (8 a)

Cream white solid; Chemical formula: C_10_H_14_N_6_; Yield; 62.25 %, Mol wt: 218.26 gmol^−1^.

^**1**^ **H NMR** (400 MHz, DMSO‐d_6_) δ 12.49 (s, 1H), 8.09 (s, 1H), 3.06 (t, *J*=5.3 Hz, 4H), 1.71 (t, *J*=5.7 Hz, 4H), 1.55 (p, *J*=3.3 Hz, 2H).

^**13**^ **C NMR** (101 MHz, DMSO‐d_6_) δ 158.13, 156.51, 155.98, 153.55, 92.70, 52.41, 25.50, 24.32.

**HRMS**: (ESI^+^‐MS, *m*/*z*) calcd for C_10_H_14_N_6_ (M)^+^: 217.1202; found: 217.1205.

#### 3‐(4‐methylpiperidin‐1‐yl)‐1H‐pyrazolo[3,4–d]pyrimidin‐4‐amine (8 b)

Yellow solid; Chemical formula: C_11_H_16_N_6_; Yield; 59.19 %, Mol wt: 232.28 gmol^−1^.

^**1**^ **H NMR** (400 MHz, DMSO‐d_6_) δ 12.49 (s, 1H), 8.09 (s, 1H), 3.41 (s, 2H), 2.80–2.69 (m, 2H), 1.67 (dd, *J*=12.1, 3.1 Hz, 2H), 1.45 (dd, *J*=11.2, 5.2 Hz, 2H), 0.96 (d, *J*=6.0 Hz, 3H) (H‐4’ peak coalesces with water peak).

^**13**^ **C NMR** (101 MHz, DMSO‐d_6_) δ 158.13, 156.51, 155.98, 153.55, 92.70, 52.41, 25.50, 24.32.

**HRMS**: (ESI^+^‐MS, *m*/*z*) calcd for C_11_H_16_N_6_ (M)^+^: 231.1358; found: 231.1350.

#### 3‐Morpholino‐1H‐Pyrazolo[3,4–d]pyrimidin‐4‐Amine (8 c)

Brown solid; Chemical formula: C_9_H_12_N_6_O; Yield; 79.88 %, Mol wt: 220.23 gmol^−1^.

^**1**^ **H NMR** (400 MHz, DMSO‐d_6_) δ 12.60 (s, 1H), 8.10 (s, 1H), 6.83 (s, 2H), 3.80 (t, *J*=4.7 Hz, 4H), 3.08 (t, *J*=4.7 Hz, 4H).

^**13**^ **C NMR** (101 MHz, DMSO‐d_6_) δ 158.08, 156.56, 156.15, 152.68, 92.52, 66.09, 51.70.

**HRMS**: (ESI^+^‐MS, *m*/*z*) calcd for C_9_H_12_N_6_O (M)^+^: 219.0994; found: 219.0991.

#### 3‐(4‐Methylpiperazin‐1‐yl)‐1H‐Pyrazolo[3,4–d]pyrimidin‐4‐Amine (8 d)

Cream solid; Chemical formula: C_10_H_15_N_7_; Yield; 62.16 %, Mol wt: 233.27 gmol^−1^.

^**1**^ **H NMR** (400 MHz, DMSO‐d_6_) δ 12.60 (s, 1H), 8.10 (s, 1H), 3.90 (s, 2H), 3.11 (t, *J*=4.8 Hz, 4H), 2.58 (t, *J*=4.8 Hz, 4H), 2.27 (s, 3H).

^**13**^ **C NMR** (101 MHz, DMSO‐d_6_) δ 158.08, 156.57, 156.06, 152.63, 92.59, 54.29, 50.89, 46.04.

**HRMS**: (ESI^+^‐MS, *m*/*z*) calcd for C_10_H_15_N_7_ (M)^+^: 234.1467; found: 234.1462.

#### 3‐(4‐Ethylpiperazin‐1‐yl)‐1H‐Pyrazolo[3,4–d]pyrimidin‐4‐Amine (8 e)

Light yellow solid; Chemical formula: C_10_H_15_N_7_; Yield; 52.64 %, Mol wt: 247.30 gmol^−1^.

^**1**^ **H NMR** (400 MHz, DMSO‐d_6_) δ 12.52 (s, 1H), 8.13–8.08 (m, 1H), 6.68 (s, 2H), 3.10 (d, *J*=5.7 Hz, 4H), 2.58 (t, *J*=4.7 Hz, 4H), 2.40 (q, *J*=7.4 Hz, 2H), 1.03 (t, *J*=7.2 Hz, 3H).

^**13**^ **C NMR** (101 MHz, DMSO‐d_6_) δ 158.10, 156.55, 156.09, 152.76, 92.66, 52.22, 52.13, 51.27, 12.40.

**HRMS**: (ESI^+^‐MS, *m*/*z*) calcd for C_11_H_17_N_7_ (M)^+^: 248.1624; found: 248.1618.

#### General Synthesis of Quinoline‐Pyrazolopyrimidine Hybrids 10 a–g

Compounds **4** or **8 a**–**g** (117.3 mg, 0.51 mmol) and potassium carbonate (104.7 g, 0.75 mmol, 1,5 eq) were dissolved in DMSO and stirred for 15 minutes, then 4.7‐dichloroquinoline (100.00 mg, 0.51 mmol) was added and the mixture was stirred under reflux at 110 °C for 12 hours. After cooling to r.t the mixture was concentrated on silica gel and purified by column chromatography using ethyl acetate‐methanol eluent to afford the quinoline hybrids in 28.47‐ 67.49 % yield.

#### 1‐(7‐Chloroquinolin‐4‐yl)‐1H‐Pyrazolo[3,4–d]pyrimidin‐4‐Amine (10 a)

Yellow solid; Chemical formula: C_14_H_9_ClN_6_; Yield; 54.28 %, Mol wt: 296.71 gmol^−1^ M.p: 206.9‐207.5 °C.

^**1**^ **H NMR** (600 MHz, DMSO‐d_6_) δ 9.11 (d, *J*=4.7 Hz, 1H, H‐2”), 8.56 (s, 1H, H‐3), 8.26 (s, 1H, H‐6), 8.23 (d, *J*=2.2 Hz, 1H, H‐8”), 8.13 (d, *J*=9.1 Hz, 1H, H‐5”), 7.89 (d, *J*=4.7 Hz, 1H, H‐3”), 7.69 (dd, *J*=9.1, 2.2 Hz, 1H, H‐6”).

^**13**^ **C NMR** (151 MHz, DMSO‐d_6_) δ 158.91 (C9), 157.58 (C6), 155.58 (C‐4), 152.51 (C‐2”), 150.15 (C‐8”a), 141.89 (C‐4”), 136.20 (C‐3), 135.25 (C‐7”), 128.39 (C6”), 128.28 (C‐8”), 127.28 (C‐5”), 121.91 (C‐4a), 118.62 (C‐3”), 101.38 (C‐8).

**HRMS**: (ESI^+^‐MS, *m*/*z*) calcd for C_14_H_9_ClN_6_ (M)^+^: 297.0655; found: 297.0652.

#### 1‐(7‐Chloroquinolin‐4‐yl)‐3‐(piperidin‐1‐yl)‐1H‐Pyrazolo[3,4–d]pyrimidin‐4‐Amine (10 b)

Yellow solid; Chemical formula: C_20_H_20_ClN_7_; Yield; 49.67 %, Mol wt: 379.85gmol^−1^ M.p: 212.8‐215.3 °C.

^**1**^ **H NMR** (400 MHz, DMSO‐d_6_) δ 9.03 (d, *J*=4.8 Hz, 1H), 8.36 (d, *J*=9.1 Hz, 1H), 8.24 (s, 1H), 8.17 (d, *J*=2.2 Hz, 1H), 7.89 (d, *J*=4.8 Hz, 1H), 7.67 (dd, *J*=9.1, 2.2 Hz, 1H), 3.23 (t, *J*=5.4 Hz, 4H), 1.76 (t, *J*=5.8 Hz, 4H), 1.60 (p, *J*=5.7 Hz, 2H).

^**13**^ **C NMR** (101 MHz, DMSO‐d_6_) δ 158.58, 157.53, 156.68, 155.34, 152.29, 150.30, 141.99, 134.99, 128.30, 127.83, 127.76, 121.71, 117.66, 94.50, 51.81, 25.15, 24.24.

**HRMS**: (ESI^+^‐MS, *m*/*z*) calcd for C_20_H_20_ClN_7_ (M)^+^: 380.1390; found: 380.1391.

##### 1‐(7‐chloroquinolin‐4‐yl)‐3‐(4‐methylpiperidin‐1‐yl)‐1H‐pyrazolo[3,4–d]pyrimidin‐4‐amine (10 c)

Light yellow solid; Chemical formula: C_20_H_20_ClN_7_; Yield; 61.21 %, Mol wt: 393.87 gmol^−1^ M.p: 239.1‐243.5 °C.

^**1**^ **H NMR** (400 MHz, DMSO‐d_6_) δ 9.04 (d, *J*=4.8 Hz, 1H), 8.36 (d, *J*=9.1 Hz, 1H), 8.24 (s, 1H), 8.18 (d, *J*=2.2 Hz, 1H), 7.89 (d, *J*=4.8 Hz, 1H), 7.67 (dd, *J*=9.1, 2.2 Hz, 1H), 1.72 (d, *J*=11.0 Hz, 2H), 1.57–1.48 (m, 2H), 1.27–1.21 (m, 4H), 0.98 (d, *J*=5.4 Hz, 3H).

^**13**^ **C NMR** (101 MHz, DMSO‐d_6_) δ 158.57, 157.54, 156.67, 155.18, 152.31, 150.29, 142.00, 135.01, 128.30, 127.82, 127.78, 121.72, 117.70, 94.52, 51.17, 33.40, 22.32.

#### 1‐(7‐Chloroquinolin‐4‐yl)‐3‐Morpholino‐1H‐Pyrazolo[3,4–d]pyrimidin‐4‐Amine (10 d)

Light brown solid; Chemical formula: C_18_H_16_ClN_7_O; Yield; 67.49 %, Mol wt: 381.82 gmol^−1^ M.p: 210.8‐214.3 °C.

^**1**^ **H NMR** (400 MHz, DMSO‐d_6_) δ 9.12 (d, *J*=4.7 Hz, 1H), 8.42 (d, *J*=9.1 Hz, 1H), 8.32 (s, 1H), 8.26 (d, *J*=2.2 Hz, 1H), 7.97 (d, *J*=4.8 Hz, 1H), 7.74 (dd, *J*=9.2, 2.3 Hz, 1H), 3.94 (t, *J*=4.5 Hz, 4H), 3.32 (t, *J*=4.6 Hz, 4H).

^**13**^ **C NMR** (101 MHz, DMSO‐d_6_) δ 158.50, 157.58, 156.78, 154.59, 152.33, 150.28, 141.90, 135.03, 127.86, 127.81, 121.73, 117.87, 94.24, 65.81, 51.09.

**HRMS**: (ESI^+^‐MS, *m*/*z*) calcd for C_18_H_16_ClN_7_O (M)^+^: 380.1027; found: 380.1023.

#### 1‐(7‐Chloroquinolin‐4‐yl)‐3‐(4‐Methylpiperazin‐1‐yl)‐1H‐Pyrazolo[3,4–d]pyrimidin‐4‐Amine (10 e)

Light yellow solid; Chemical formula: C_19_H_19_ClN_8_; Yield; 28.47 %, Mol wt: 394.86 gmol^−1^ M.p: 205.6‐208.5 °C.

**1H NMR** (400 MHz, DMSO‐d_6_) δ 9.04 (d, J=4.8 Hz, 1H, H‐2”), 8.34 (d, J=9.1 Hz, 1H, H‐5”), 8.24 (s, 1H, H‐6), 8.17 (d, J=2.2 Hz, 1H, H‐8”), 7.89 (d, J=4.8 Hz, 1H, H‐3”), 7.66 (dd, J=9.1, 2.2 Hz, 1H, H‐6”), 7.14 (s, 2H, H‐4a), 3.31–3.24 (m, 4H, H‐2’,6’H‐2’&7’), 2.59 (dd, J=7.4, 2.5 Hz, 4H, H‐3’,5’H‐3’,6’), 2.25 (d, J=5.2 Hz, 3H, H‐7’H‐5’).

**13 C NMR** (101 MHz, DMSO‐d_6_) δ 158.50 (C‐3), 157.58 (C‐6), 156.68 (C‐9), 154.64 (C‐4), 152.32 (C‐2’), 150.23 (C‐8”a), 141.90 (C‐4”), 135.04 (C‐7”), 128.26 (C‐8”), 127.84 (C‐5), 127.78 (C‐6”), 121.70 (C‐4”a), 117.80 (C‐3”), 94.37 (C‐8), 54.09 (C‐3’,6’), 50.53 (C‐2’,7), 46.26 (C‐5’).

**HRMS**: (ESI^+^‐MS, *m*/*z*) calcd for C_19_H_19_ClN_8_ (M)^+^: 393.1343; found: 393.1347.

#### 1‐(7‐Chloroquinolin‐4‐yl)‐3‐(4‐Ethylpiperazin‐1‐yl)‐1H‐Pyrazolo[3,4–d]pyrimidin‐4‐Amine (10 f)

Light yellow solid; Chemical formula: C_20_H_21_ClN_8_; Yield; 40.97 %, Mol wt: 408.89 gmol^−1^ M.p: 204.0‐208.7 °C.

^**1**^ **H NMR** (400 MHz, DMSO‐d_6_) δ 9.04 (d, *J*=4.8 Hz, 1H), 8.35 (d, *J*=9.2 Hz, 1H), 8.24 (s, 1H), 8.18 (d, *J*=2.2 Hz, 1H), 7.89 (d, *J*=4.8 Hz, 1H), 7.66 (dd, *J*=9.1, 2.3 Hz, 1H), 3.27 (t, *J*=4.8 Hz, 4H), 2.64 (t, *J*=4.7 Hz, 4H), 2.42 (q, *J*=7.1 Hz, 2H), 1.04 (t, *J*=7.1 Hz, 3H).

^**13**^ **C NMR** (101 MHz, DMSO‐d_6_) δ 158.51, 157.58, 156.70, 154.63, 152.31, 150.27, 141.88, 135.00, 128.30, 127.84, 127.81, 121.68, 117.76, 94.34, 52.14, 51.81, 50.65, 12.38.

**HRMS**: (ESI^+^‐MS, *m*/*z*) calcd for C_20_H_21_ClN_8_ (M)^+^: 407.1499; found: 407.1498.

#### 2‐(4‐(4‐Amino‐1‐(7‐Chloroquinolin‐4‐yl)‐1H‐Pyrazolo[3,4–d]pyrimidin‐3‐yl)piperazin‐1‐yl)ethanol (10 g)

Light brown solid; Chemical formula: C_20_H_21_ClN_8_O; Yield; 38.39 %, Mol wt: 424.89 gmol^−1^ M.p: 225.8‐231.4 °C.

^**1**^ **H NMR** (600 MHz, DMSO‐d_6_ ) δ 9.07 (d, *J*=4.8 Hz, 1H), 8.38 (d, *J*=9.2 Hz, 1H), 8.27 (s, 1H), 8.21 (d, *J*=2.2 Hz, 1H), 7.92 (d, *J*=4.8 Hz, 1H), 7.70 (dd, *J*=9.1, 2.0 Hz, 1H), 3.58 (t, *J*=6.3 Hz, 2H), 3.29 (d, *J*=6.1 Hz, 4H), 2.74–2.68 (m, 4H), 2.50 (dd, *J*=14.0, 6.8 Hz, 2H).^**13**^ **C NMR** (151 MHz, DMSO‐d_6_) δ 161.60, 158.53, 157.58, 156.74, 154.65 (d, *J*=7.2 Hz), 152.30, 150.29, 141.93, 135.02, 128.30, 127.80, 121.73, 117.76, 94.41, 52.68, 52.15, 50.72, 50.67.

**HRMS**: (ESI^+^‐MS, *m*/*z*) calcd for C_20_H_21_ClN_8_O (M)^+^: 423.1449; found: 423.1440.

### General Synthesis of Quinoline‐4‐Amine Hybrids 12 a–h

A solution of 4,7‐dichloroquinoline (200 mg, 1.00 mmol) in isopropyl alcohol was stirred at r.t for 5 minutes then appropriate *para*‐substituted aniline (128.80 mg, 1.00 mmol) was added and stirring continued for 4 hours. The resulting precipitate was filtered and dried under vacuum to afford quinoline hybrids **12 a**‐**h** in 68.88‐85.69 % yield.

#### 7‐Chloro‐N‐Phenylquinolin‐4‐Amine (12 a)

Lemon yellow solid; Chemical formula: C_15_H_11_ClN_2_; Yield; 71.35 %, Mol wt: 254.71 gmol^−1^ M.p: >250 °C.

^**1**^ **H NMR** (600 MHz, DMSO‐d_6_) δ 11.34 (s, 1H), 8.96 (d, *J*=9.1 Hz, 1H), 8.52 (d, *J*=7.0 Hz, 1H), 8.21 (d, *J*=2.1 Hz, 1H), 7.85 (dd, *J*=9.1, 2.1 Hz, 1H), 7.58 (dd, *J*=8.3, 7.3 Hz, 2H), 7.50 (dd, *J*=8.5, 1.3 Hz, 2H), 7.44 (tt, *J*=7.0, 1.2 Hz, 1H), 6.77 (d, *J*=7.0 Hz, 1H).^13^C NMR (151 MHz, DMSO‐d_6_) δ 155.36, 143.76, 139.60, 138.81, 137.48, 130.42, 128.05, 127.75, 126.79, 125.93, 119.65, 116.44.

**HRMS**: (ESI^+^‐MS, *m*/*z*) calcd for C_15_H_11_ClN_2_ (M)^+^: 253.0533; found: 253.0531.

#### 7‐Chloro‐N‐(4‐Fluorophenyl)quinolin‐4‐Amine (12 b)

Bright yellow solid; Chemical formula: C_15_H_10_ClFN_2_; Yield; 68.88 %, Mol wt: 272.70 gmol^−1 1^ M.p: >250 °C.

^**1**^ **H NMR** (400 MHz, DMSO‐d_6_) δ 11.29 (s, 1H), 8.94 (d, *J*=9.1 Hz, 1H), 8.52 (d, *J*=7.0 Hz, 1H), 8.21 (d, *J*=2.2 Hz, 1H), 7.85 (dd, *J*=9.1, 2.1 Hz, 1H), 7.55 (ddd, *J*=8.4, 5.3, 2.8 Hz, 2H), 7.42 (t, *J*=8.8 Hz, 2H), 6.72 (d, *J*=7.0 Hz, 1H).

^**13**^ **C NMR** (101 MHz, DMSO‐d_6_) δ 155.50, 144.16, 139.80, 138.78, 133.83, 128.36 (d, *J*=8.8 Hz), 127.81, 126.55, 119.95, 117.33 (d, *J*=22.7 Hz), 116.40, 100.75, 100.72.

**HRMS**: (ESI^+^‐MS, *m*/*z*) calcd for C_15_H_10_ClFN_2_ (M)^+^: 273.0595; found: 273.0596.

#### 7‐Chloro‐N‐(4‐Chlorophenyl)quinolin‐4‐Amine (12 c)

Bright yellow solid; Chemical formula: C_15_H_10_C_l2_N_2_; Yield; 75.61 %, Mol wt: 289.16 gmol^−1^ M.p: >250 °C.

^**1**^ **H NMR** (400 MHz, DMSO‐d_6_) δ 11.25 (s, 1H), 8.92 (dd, *J*=9.1, 1.2 Hz, 1H), 8.54 (dd, *J*=6.9, 1.4 Hz, 1H), 8.20 (t, *J*=1.7 Hz, 1H), 7.85 (dt, *J*=9.2, 1.8 Hz, 1H), 7.62 (dd, *J*=8.7, 1.6 Hz, 2H), 7.54 (dd, *J*=8.7, 1.5 Hz, 2H), 6.84 (dd, *J*=7.0, 1.4 Hz, 1H).

^**13**^ **C NMR** (101 MHz, DMSO‐d_6_) δ 154.65, 144.76, 140.45, 138.55, 136.80, 131.77, 130.36, 127.74, 127.42, 126.54, 120.47, 116.75, 101.13.

**HRMS**: (ESI^+^‐MS, *m*/*z*) calcd for C_15_H_10_Cl_2_N_2_ (M)^+^: 289.0299; found: 289.0297.

#### N‐(4‐Bromophenyl)‐7‐Chloroquinolin‐4‐Amine (12 d)

Bright yellow solid; Chemical formula: C_15_H_10_BrClN_2_; Yield; 82.14 %, Mol wt: 333.61 gmol^−1^ M.p: >250 °C.

^**1**^ **H NMR** (400 MHz, DMSO‐d_6_) δ 8.90 (d, *J*=9.1 Hz, 1H), 8.53 (d, *J*=6.8 Hz, 1H), 8.18 (d, *J*=2.2 Hz, 1H), 7.82 (dd, *J*=9.1, 2.2 Hz, 1H), 7.73 (d, *J*=8.7 Hz, 2H), 7.46 (d, *J*=8.7 Hz, 2H), 6.86 (d, *J*=6.8 Hz, 1H).

^**13**^ **C NMR** (101 MHz, DMSO‐d_6_) δ 152.50, 147.12, 138.17, 137.34, 133.11, 127.12, 126.71, 126.01, 122.84, 118.78, 101.69.

**HRMS**: (ESI^+^‐MS, *m*/*z*) calcd for C_15_H_10_BrClN_2_ (M)^+^: 332.9794; found: 332.9789.

#### 7‐Chloro‐N‐(p–tolyl)quinolin‐4‐Amine (12 e)

Yellow solid; Chemical formula: C_16_H_13_ClN_2_; Yield; 73.54 %, Mol wt: 268.74 gmol^−1^ M.p: >250 °C.

^**1**^ **H NMR** (400 MHz, DMSO‐d_6_) δ 11.17 (s, 1H), 8.92 (d, *J*=9.1 Hz, 1H), 8.48 (d, *J*=7.0 Hz, 1H), 8.19 (d, *J*=2.1 Hz, 1H), 7.82 (dd, *J*=9.1, 2.2 Hz, 1H), 7.36 (s, 4H), 6.72 (d, *J*=7.0 Hz, 1H), 2.38 (s, 3H).

^**13**^ **C NMR** (101 MHz, DMSO‐d_6_) δ 155.02, 144.38, 140.34, 138.47, 137.41, 135.00, 130.87, 127.56, 126.47, 125.72, 120.31, 116.49, 100.68, 21.16.

**HRMS**: (ESI^+^‐MS, *m*/*z*) calcd for C_16_H_13_ClN_2_ (M)^+^: 269.0846; found: 269.0840.

#### 7‐Chloro‐N‐(4‐Methoxyphenyl)quinolin‐4‐Amine (12 f)

Bright yellow solid; Chemical formula: C_16_H_13_ClN_2_O; Yield; 84.53 %, Mol wt: 284.74 gmol^−1^ M.p: >250 °C.

^**1**^ **H NMR** (400 MHz, DMSO‐d_6_) δ 11.20 (s, 1H), 8.93 (d, *J*=9.1 Hz, 1H), 8.47 (d, *J*=7.0 Hz, 1H), 8.19 (d, *J*=2.2 Hz, 1H), 7.80 (dd, *J*=9.1, 2.1 Hz, 1H), 7.39 (d, *J*=8.8 Hz, 2H), 7.11 (d, *J*=8.9 Hz, 2H), 6.64 (d, *J*=7.0 Hz, 1H), 3.82 (s, 3H).

^**13**^ **C NMR** (101 MHz, DMSO‐d_6_) δ 158.89, 155.63, 143.60, 139.64, 138.63, 129.95, 127.55, 126.71, 119.65, 116.23, 115.56, 100.43, 55.94.

**HRMS**: (ESI^+^‐MS, *m*/*z*) calcd for C_16_H_13_ClN_2_O (M)^+^: 285.0795; found: 285.0792.

#### 7‐Chloro‐N‐(4‐(trifluoromethoxy)phenyl)quinolin‐4‐Amine (12 g)

Lemon solid; Chemical formula: C_16_H_10_ClF_3_N_2_O; Yield; 85.69 %, Mol wt: 338.71 gmol^−1^ M.p: >250 °C.

^**1**^ **H NMR** (600 MHz, DMSO‐d_6_) δ 11.00 (s, 1H), 8.84 (d, *J*=8.6 Hz, 1H), 8.55 (d, *J*=6.7 Hz, 1H), 8.16 (d, *J*=2.0 Hz, 1H), 7.85 (dd, *J*=9.1, 2.2 Hz, 1H), 7.63 (d, *J*=8.9 Hz, 2H), 7.56 (d, *J*=8.5 Hz, 2H), 6.88 (d, *J*=6.7 Hz, 1H).

^**13**^ **C NMR** (151 MHz, DMSO‐d_6_) δ 153.15, 146.59, 146.47, 142.64, 137.75, 137.64, 127.27, 126.67, 126.20, 122.97, 122.26, 121.43, 119.73, 117.28, 101.55.

**HRMS**: (ESI^+^‐MS, *m*/*z*) calcd for C_16_H_10_ClF_3_N_2_O (M)^+^: 339.0512; found: 339.0501.

#### 7‐Chloro‐N‐(4‐Nitrophenyl)quinolin‐4‐Amine (12 h)

Yellow solid; Chemical formula: C_15_H_10_ClN_3_O_2_; Yield; 78.16 %, Mol wt: 299.71 gmol^−1^ M.p: >250 °C.

^**1**^ **H NMR** (400 MHz, DMSO‐d_6_) δ 8.88 (d, *J*=4.7 Hz, 1H), 8.22 (d, *J*=9.0 Hz, 1H), 8.17 (d, *J*=2.1 Hz, 1H), 7.94 (d, *J*=9.1 Hz, 1H), 7.83–7.76 (m, 3H), 6.72 (s, 1H), 6.60 (d, *J*=9.2 Hz, 1H).

^**13**^ **C NMR** (101 MHz, DMSO‐d_6_) δ 156.17, 152.41, 149.31, 141.81, 135.88, 129.22, 128.63, 126.85, 126.33, 124.78, 122.55, 112.84.

### In vitro Biological Evaluation

#### α‐Glucosidase Inhibition

The α‐glucosidase inhibitory potency of each compound was determined using the described protocol by Ademiluyi et al..^[46]^ 0.2 mL aliquot of different concentrations of acarbose or test compounds (62.5‐500 μM) was added to 0.4 mL yeast α‐glucosidase (1 U/mL) solution in 0.1 M phosphate buffer (pH 6.8). The reaction was incubated at 37 °C for 10 minutes before adding 0.2 mL of *para*‐nitrophenyl‐D‐glucopyranoside solution (5000 μM). After further incubation for another 20 minutes, the absorbance of the resulting solution was taken at 405 nm against a blank solution without the test inhibitors.

$$\%\;\alpha \text{-}\mathrm{Glucosidase}\;\mathrm{inhibition}=\left(1-\frac{Test\;sample\;Abs\;}{Blank\;Abs}\right)\times 100$$

#### α‐Amylase Enzyme Inhibition

The compounds’ ability to inhibit α‐amylase was estimated using the method of Ibitoye et al.^[47]^ with minor modifications. 0.2 mL of each compound or acarbose at concentration 62.5‐500 μM was added to 0.2 mL solution of porcine pancreatic amylase solution containing 0.02 M phosphate buffer (pH 6.9) and NaCl (6 μM). The mixture was equilibrated for 10 minutes at r.t before adding 1 % starch solution (0.4 mL). This step was followed by further incubation for 15 minutes under the previous condition before the mixture reaction was terminated by boiling for 10 minutes with dinitrosalicylate reagent (1000 μL). After cooling, the solution was diluted with 5000 μL distilled water before the absorbance was measured at 540 nm against a blank solution lacking the test compounds or acarbose.

$$\%\;\alpha \text{-}\mathrm{Amylase}\;\mathrm{inhibition}=\left(1-\frac{Test\;sample\;Abs\;}{Blank\;Abs}\right)\times 100$$

### Antioxidant Activity

#### 2,2′‐Diphenyl‐1‐Picrylhydrazyl (DPPH) Radical Scavenging Activity

The capacity of the chemical compounds to decolourize the purple colour of DPPH solution was investigated following a modified procedure of Turkoglu et al..^[48]^ 1000 μL aliquot of prepared solution of each compound (62.5–500 μM) and DPPH (300 μM in methanol) were added together. The solution was mixed gently before equilibration in a dark compartment for 30 minutes at 25 °C. The absorbance (Abs) of the resulting mixture was read 517 nm against a blank solution lacking the test samples. The DPPH radical scavenging ability of each compound was estimated using the expression below:

$$\%\;\mathrm{DPPH}\;\mathrm{scavenging}=\left(1-\frac{Test\;sample\;Abs\;}{Blank\;Abs}\right)\times 100$$

#### Nitric Oxide (NO) Scavenging Activity

The NO radical mop‐up ability of the compounds was evaluated using Kurian et al.^[49]^ previous protocol. 0.5 ml solution of 62.5–500 μM concentrations of each compound was added to 10 mM of sodium nitroprusside (0.25 mL) phosphate‐buffered saline (pH 7.4). After 2 hr equilibration at 37 °C, 0.250 mL of Griess reagent was added, and the absorbance of the coloured solution produced was measured at 545 nm against a blank solution lacking the test samples. Subsequently, the % NO scavenging antioxidant capacity of the compounds was expressed as:

$$\%\;\mathrm{Nitric}\;\mathrm{oxide}\;\mathrm{scavenging}=\left(1-\frac{Test\;sample\;Abs\;}{Blank\;Abs}\right)\times 100$$

#### Molecular Docking Protocols

The *in silico* calculations were performed with Schrödinger suite (2022‐1 release) using OPLS4 forcefield.

#### Protein Preparation

The homology model was prepared according to our previous work.^[50]^ The enzyme model was prepared using the protein preparation module by adding missing hydrogens, adding missing side chains and loops using Prime,^[51]^ deleting water molecules that are more that 5 Å away from the binding cavity, generating tautomer's and heteroatomic states at pH 7.0±2.0 with Epik.^[52]^ This was followed by assigning the protonation of Asp, His and Asn to favor hydrogen bonding. Hydrogen bond network optimization for the entire system was performed followed by minimization to an RMSD of 0.3 Å. The binding site was lastly subjected to energy minimization with Prime.

#### Ligand Preparation

Ligand structures were drawn with ChemDraw and exported to Maestro. LigPrep module was used to generate 3D models of the lowest energy conformation.

#### Induced‐Fit Docking and MMGBSA Binding Free Energy

Induced fit‐docking module was used to dock the prepared ligands into the active site. The workflow included defining the ligand box by selecting a box using the centroid of selected residues, setting the box size to be <20 Å and performing conformational sampling to 2.5 kcal.mol^−1^energy window. Subsequently, the ligands were docked with glide and formed poses side chains were further minimized with Prime. Poses with an energy of 30 kcal.mol^−1^ were further redocked and the best 10 poses were further processed and analyzed. The selected best pose of each protein‐ligand complex based on the docking score, IFD scores, glide emodel and glide energy was used for Prime Molecular Mechanics‐Generalized Borne Surface Area (MMGBSA) to calculate the binding free energy (ΔG_bind_) of the protein‐ligand complex.

#### In Vitro Anti‐Mycobacterial Activity

The anti‐*Mtb* activities of the target compounds were established using a previously described method.^[53]^ Briefly, a 10 mL culture of the *Mtb* pMSp12::GFP reporter strain was grown to an optical density (OD_600_) of 0.6‐0.7 in Middlebrook 7H9 medium supplemented with either 0.03 % casitone (CAS), 0.4 % glucose, and 0.05 % tyloxapol, or 0.03 % albumin, 0.4 % glucose, and 0.05 % Tween 80. The cultures were diluted 1 : 500, and 50 μL of *Mtb*‐containing medium was introduced into each well of a 96‐well plate, followed by the addition of target compounds at concentration range between 0.244‐125 μM. The plates were sealed, incubated at 37 °C with 5 % CO_2_ and humidification. Rifampicin (RIF) (MIC_90_) and 5 % DMSO were used as the minimum, and maximum growth controls, respectively. On day 7 or day 14 following incubation, fluorescence readings were obtained using a Spectramax i3x Plate reader (Serial no. 36370 3271) running Softmax® Pro 6 software (Version 6.5.1, Serial no. 1278552768867612530), Molecular Devices Corporation 1311 Orleans Drive Sunnyvale, California 94089. The onboard Fluorescent Intensity‐Endpoint protocol was used in conjunction with the following wavelength filters: Excitation, 485 nm; Emission, 520 nm. The Softmax ® Pro 6 4‐parameter curve fit protocol was used to generate a calculated MIC_90_. Raw RFU data were normalised to the minimum and maximum inhibition controls to generate a dose response curve (% inhibition), using the Levenberg‐Marquardt damped least‐squares (DLS) method, from which the MIC_90_ was calculated. The lowest concentration of drug that inhibited 90 % of growth of the bacterial population was considered to be the MIC_90_.

## Conflict of Interests

The authors declare no conflict of interest.

1

## Data Availability

The data that support the findings of this study are available in the supplementary material of this article.
